# A pilot study protocol of a relational coordination training intervention among healthcare professionals in an Army medical center

**DOI:** 10.1186/s40814-025-01596-7

**Published:** 2025-03-04

**Authors:** Sherita House, Susan M. Perkins, Melissa Miller, Tanekkia Taylor-Clark, Robin Newhouse

**Affiliations:** 1 University of North Carolina at Greensboro, 1007 Walker Ave., Greensboro, NC 27402 USA; 2 Indiana University School of Medicine, 410 W. 10Th Street, Suite 3000, Indianapolis, IN 46202 USA; 3 Center for Nursing Science and Clinical Inquiry, Tripler Army Medical Center, 1 Jarrett White Road, Honolulu, HI 96818 USA; 4 Center for Nursing Science and Clinical Inquiry, Womack Army Medical Center, 2817 Reilly Road, Fort Liberty, NC 28310 USA; 5 Indiana University School of Nursing, 600 Barnhill Drive, NU 130, Indianapolis, IN 46202 USA

**Keywords:** Relational coordination, Intervention, Job satisfaction, Quality of care, Intent to stay

## Abstract

**Background:**

As patient care becomes more complex, high-quality communication and relationships among healthcare professionals are critical to coordinating care. Relational coordination (RC), a process of high-quality communication supported by shared goals, shared knowledge, and mutual respect, is positively associated with better patient (e.g., quality of care) and staff (e.g., job satisfaction, and retention) outcomes. A few researchers have found that communication skills training improves RC in civilian hospitals. However, researchers have not tested the feasibility of conducting communication skills training based on the RC framework among healthcare professionals in military hospitals. To address this gap, we propose conducting an RC training intervention in a military hospital. The primary aim of the proposed pilot study is to determine the feasibility (e.g., recruitment, retention, and completion rates) of conducting an RC training intervention in an Army medical center. The secondary aim is to explore the acceptability and usability of the RC training intervention. We will also explore changes in RC, quality of care, job satisfaction, and intent to stay among participants following the RC training intervention.

**Methods:**

A single-group feasibility study will be conducted among nurses and physicians from three units (intensive care unit, medical-surgical, and labor and delivery unit). A convenience sample of licensed practical nurses (LPNs), registered nurses (RNs), resident physicians, and physicians from the participating units will be invited to complete a 1-h RC training intervention once a month for 3 months. Participants will complete RC, quality of care, job satisfaction, and intent to stay measures at baseline and 2 weeks after each RC training intervention session. To assess the feasibility of conducting an RC training intervention, we will examine recruitment/retention rates, intervention session completion rates, and survey measure completion rates. Acceptability will be assessed qualitatively through focus group interviews, and results will be used to refine the intervention and determine if the selected measures align with participant experiences. For our secondary aim, we will explore the acceptability of the RC training intervention through focus group interviews. We will also explore changes in outcome measures using descriptive statistics with 95% confidence intervals.

**Discussion:**

Findings will establish the feasibility and acceptability of conducting an RC intervention in a military hospital and inform refinement of the intervention and study procedures prior to conducting a larger randomized controlled trial to establish efficacy.

**Supplementary Information:**

The online version contains supplementary material available at 10.1186/s40814-025-01596-7.

## Background

Patient care is becoming more complex, as patients are presenting with uncertain diagnoses and multiple co-morbidities [1, 2]. This is a critical problem within the healthcare system as healthcare professionals are challenged to meet the increased time constraints and demands that accompany caring for complex patients. As patient care becomes more complex, high-quality communication and relationships among healthcare professionals are critical to coordinating care [3–6]. While uncoordinated care is a problem in civilian healthcare settings, military healthcare settings may encounter additional barriers to coordinating care because of the unique hierarchical ranking structures that exist among healthcare professionals in military hospitals [7, 8]. Understanding RC in a military context poses a unique opportunity to examine a complex organizational structure due to the presence of clear authority gradients and differences in military rank. Additionally, explicit, formal power differences exist between enlisted service members, commissioned officers, and members in different ranks within each personnel structure. These hierarchical ranking structures create power differences among healthcare professionals in different ranks. Power differences among members in a workgroup can negatively influence communication and relationships [9], leading to lower job satisfaction and retention.

Relational coordination (RC), defined as “a mutually reinforcing process of communicating and relating for the purpose of task integration within and between roles,” ([10] p. 301) is a unique way to coordinate care among healthcare professionals involved in complex work processes. RC encompasses four communication dimensions (frequent, timely, accurate, and problem-solving communication) and three relationship dimensions (shared goals, shared knowledge, and mutual respect) [10, 11] See Table 1.

**Table 1:** Dimensions and definitions of relational coordination [10, 11]

Seven relational coordination dimensions	Definitions
Frequent communication	How often professionals communicate regarding their work process
Timely communication	How soon professionals report significant information regarding a work process
Accurate communication	Preciseness of information communicated between professionals regarding a work process
Problem-solving communication	The extent to which professionals seek solutions instead of placing blame when problems occur
Shared goals	Understanding how the work of each professional fits together with the work of other professionals in the same work process
Shared knowledge	An understanding of the role of other professionals, which includes an awareness of who needs to know what and why and when
Mutual respect	Valuing the work of other professionals

Researchers have sought to address the problem of coordinating patient care for complex patients through RC and communication skills training [12–15]. In observational studies, RC has been associated with improved patient outcomes (e.g., quality of care) [16] and better staff outcomes (e.g., higher job satisfaction and retention) among nurses and physicians in civilian hospitals [13, 17–20]. A few researchers have also conducted communication skills training to improve RC among civilian healthcare professionals, and in these studies, patient outcomes improved [14, 15]. Fettig et al. [14] found that communication skills training improved RC about goals of care among nurses, physicians, social workers, and chaplains in an intensive care unit. Blakeney et al. [15] conducted a 4-year longitudinal study consisting of various training interventions (leadership workshops, interprofessional team training, and structured bedside rounds) to improve RC among healthcare professionals in a heart failure unit. Blakeney et al. [15] found that these interventions significantly improved RC scores (*p* = 0.0001) from baseline to year 4.

Although these studies show significant positive results regarding communication skills training to improve RC [14, 15], additional research is needed to determine the feasibility and acceptability of conducting RC training interventions. Moreover, the current literature is limited to civilian healthcare settings. Given that communication skills training improved RC in civilian hospitals [14, 15], we believe communication skills training will improve RC and other outcomes—such as quality of care, job satisfaction, and intent to stay—in unique settings such as military hospitals. Thus, we propose conducting communication skills training based on the RC framework in a military hospital. The primary aim of the proposed pilot study is to determine the feasibility (e.g., recruitment, retention, RC training completion rates, and survey completion rates) of conducting an RC training intervention in an Army medical center. The secondary aim is to explore the acceptability and usability of conducting an RC training intervention. We will also explore group-level changes in RC and individual-level changes in quality of care, job satisfaction, and intent to stay among nurses and physicians following an RC training intervention. We will explore the following research questions: (1) what percent of eligible participants consent to participate in the study? (2) What percent of intervention sessions do participants complete? (3) What percent of survey measures do participants complete? and (4) What are the nurses’ and physicians’ perspectives about the acceptability and usability of the RC training intervention for improving their relationships and communication on their unit, quality of care, job satisfaction, and intent to stay? Given the significant results between RC and patient and staff outcomes in prior studies, we expect that an RC training intervention will improve the quality of care, job satisfaction, and intent to stay in our study.

## Methods

### Design, setting, and ethics

This single-group feasibility study will be an extension of a previous study exploring RC, job satisfaction, and intent to stay [21]. We conducted a secondary analysis and found that participants from the intensive care, medical-surgical, and labor and delivery unit in an Army medical center reported lower job satisfaction and intent to stay. Thus, we will invite healthcare professionals from these units to participate in an RC training intervention. Our reporting follows the SPIRIT guidelines (see SPIRIT checklist in supplemental files). The Indiana University Institutional Review Board approved this study (Approval number 15936). Any significant protocol modifications will be communicated to participants and the institutional review board prior to implementing any changes.

### Sampling and recruitment

This is a feasibility study and is not a priori designed to detect clinically meaningful changes in this pilot stage. Three units will be invited to participate in this study. All units are expected to participate, with approximately twelve participants from each unit enrolling (*n* = 36) and six participants from each unit completing (*n* = 18, 50%). With a sample size of 36 enrolling, a two-sided 95% exact (Clopper-Pearson) confidence interval would have a margin of error of 17% when the completion rate is 50% [22]. Potential participants will be identified using the protocol inclusion and exclusion criteria. The principal investigator will conduct virtual meetings with nurse managers, physician leaders, and eligible participants and provide a detailed overview of the study. The principal investigator will also email a detailed overview of the study, the voluntary nature of participation, the consent process, and the assurance that participant responses will remain confidential to eligible participants. The research team will place flyers on participating units and send a follow-up email to eligible participants to inform them about the study and how to contact the research team if they desire to participate. The primary investigator will obtain verbal consent from participants prior to beginning the study (e.g., surveys, RC training intervention, focus group interviews). A five-dollar coffee card will be offered at each data collection time point to incentivize participants to complete the surveys at each data collection time point.

### Inclusion and exclusion criteria

Participants who meet the following inclusion criteria will be invited to participate in the study: (1) licensed practical nurses; (2) registered nurses; (3) physician residents; (4) physicians; (5) able to read and understand the English language; (6) employed at least 3 months at the study site; and (7) speaks and understands the English language. While the participants must be able to speak and understand the English language, our training will be inclusive and sensitive to diversity (e.g., we will include participants who speak English as a second or additional language). Exclusion criteria will be healthcare professionals who are government contractors (temporary employees).

### The RC training intervention

The RC training intervention will be conducted face-to-face after participants complete the baseline survey and RC knowledge assessment. See Additional files 1 and 2. The RC training intervention will be delivered by a member of the relational coordination collaborative group. The relational coordination collaborative group is a community of professionals from various sectors around the world who use RC in their research and practice. A 1-h RC training session will be delivered each month for 3 months, for a total of three sessions. Session one will explore the RC dimensions and evidence-based literature supporting RC and patient and staff outcomes [20, 23, 24]. Session two will be conducted 1 month later and focus on a relational mapping exercise. Relational mapping allows participants to generate new ideas for how they communicate and relate to each other regarding complex work processes [25]. Session three will be conducted 1 month after session two and will explore how to operationalize RC on their unit [26]. During the third RC training session, we will help participants identify effective strategies to improve interprofessional collaboration, quality of care, job satisfaction, and intent to stay using the dimensions of RC. See Table 2. Participants will be advised that may leave the RC intervention training sessions for any reason and at any time if they.

**Table 2:** RC training intervention overview

Relational coordination training session 1 (1 h lunch and learn)	
Objectives	• Discuss the relational coordination dimensions and organizational change model • Review relational coordination and patient and staff outcome studies
Relational coordination training session 2 (1 h lunch and learn)	
Objectives	• Participants will create color-coded relational ties between healthcare professionals involved with patient care on their unit • Identify which healthcare professionals have weak relational ties • Identify which healthcare professionals have strong relational ties • Discuss why these weak and strong relational ties exist and ways to improve weak relational ties among healthcare professionals
Relational coordination training session 3 (1 h lunch and learn)	
Objective	• Discuss how to operationalize the relational coordination dimensions • Discuss how relational coordination can be used in conjunction with TeamSTEPPS to improve patient and staff outcomes

^*^Team STEPPS (Team Strategies and Tools to Enhance Performance and Patient Safety)

### Fidelity assessment

The RC training intervention will be delivered using a standardized manual and session-specific checklists to ensure consistent delivery across facilitators and groups [27, 28]. A member of the relational coordination collaborative group will deliver the training intervention. Prior to delivering the sessions, the relational coordination collaborative group member will attend 2 h of training consisting of didactics and role-playing. Each session will be audio recorded and reviewed by two members of the research team using fidelity checklists (e.g., to determine if the training intervention was delivered as intended and consistent with the protocol). Two members of the research team will use a checklist of intervention content to independently code recordings.

### Study procedures

Data collection will occur at baseline and 2 weeks after the completion of each RC training intervention session. The surveys (RC, RC knowledge assessment, quality of care, job satisfaction, and intent to stay) will be distributed at the following time points.

Baseline (prior to the RC training intervention).

Two weeks after the RC training intervention Session 1.

Two weeks after RC training intervention Session 2.

Two weeks after the RC training intervention Session 3.

Participants will complete the survey electronically by scanning a QR code. We selected these data collection time points to explore patterns across workgroups (e.g., nurses and physicians) over time and refine subsequent RC training sessions based on survey results at each data collection time point. For example, if participants report low frequent communication scores, we can explore why participants rated this RC dimension less favorably and discuss ways to improve frequent communication during the subsequent RC training session. RC training intervention evaluations will be used to explore the acceptability of the training sessions, and we will administer the evaluation via Survey Monkey immediately after each RC training intervention session. Two weeks after the final RC training is complete, participants will be invited to participate in a 30–45-min focus group interview session, during which we will explore the acceptability of the intervention. See Table 3 for the study procedures timeline.

**Table 3:** Study procedures timeline

Activity	Quarter 3 2022	Quarter 1 2023	Quarter 2 2023	Quarter 3 2023	Quarter 4 2023	Quarter 1 2024
Prepare recruitment materials	X					
Meeting with Chief Nursing Officer and Chief Medical Officer at the study site	X					
Submit IRB Application	X					
Set up database and meeting with nurse and physician leaders of the participating units		X				
Distribute recruitment materials/letters to eligible participants			X			
Distribute baseline survey and RC knowledge assessment				X		
RC training intervention session 1				X		
RC training intervention session 2				X		
RC training intervention session 3				X		
Focus group interview session				X		
Data cleaning and entry				X	X	
Data analysis				X	X	
Dissemination of results						X

^*^Relational coordination (RC)

### Primary outcomes

We will use a mixed methods approach to establish feasibility. As such, we will collect data on feasibility as outlined below.

#### Feasibility

We will collect data on feasibility by assessing the following: (1) recruitment and retention rates; (2) the percent of eligible participants who consent to participate in the study; (3) the percent of planned intervention sessions participants complete; (4) the percent of planned measures participants complete (e.g., the number of participants who complete the survey at baseline and 2-weeks post each RC training intervention session). We will also collect data on the number of participants who participated in all three RC training intervention sessions. See Table 4 for the feasibility benchmark outcomes.

**Table 4:** Feasibility benchmark outcomes

Feasibility outcome	Feasibility benchmark of success
Recruitment rates	We will recruit at least 12 participants per unit
Retention rates	We will retain at least 50% of participants per unit (*n* = 6)
Percent of eligible participants who participate in the study	At least 50% of eligible participants per unit will consent to participate
Number of intervention sessions participants complete	Participants will complete at least two RC training intervention sessions
Percent of participants who complete the surveys at each data collection time point	At least 50% of eligible participants will complete the surveys at each data collection time point
Number of participants who participate in each RC training intervention session	At least six participants per unit will participate in each RC training intervention

*RC* relational coordination

### Secondary outcomes

The secondary aims are to explore the acceptability and usability of the RC training intervention, and changes in RC, quality of care, job satisfaction, and intent to stay among participants following the RC training intervention.

#### Acceptability

Acceptability of the intervention and study procedures will be assessed qualitatively via focus group interviews. We will use Yardley et al. [29] person-centered approach to gain an in-depth understanding of the beliefs, needs, and attitudes of the participants who participate in the RC training intervention. We selected the person-based approach to better understand how different healthcare professionals (e.g., nurses versus physicians) on different units (e.g., medical-surgical versus ICU) experience and engage with the RC training intervention. Additionally, we desire to explore which components of the RC training intervention are relevant and useful and which components of the training intervention are not [29]. Verbal consent will be obtained from participants to audio record the RC training intervention sessiocns. We will create an Excel spreadsheet to capture participant responses from the training sessions and explore similarities and differences in participant responses. The Excel files from the RC training interventions will be stored on a password-protected computer. We will explore nurses’ and physicians’ views and experiences of the RC training intervention, including what they perceive to be barriers to conducting the intervention. Focus group interviews will help the research team to (1) refine the intervention; (2) determine if the measures we selected align with the experiences of the participants; and (3) better understand the outcomes in Aim 2 (e.g., group-level changes in RC and individual-level changes in quality of care, job satisfaction, and intent to stay).

### Outcomes

This study is not powered to detect statistically significant changes in group and individual-level outcome measures. However, we will collect data on these outcomes to explore the feasibility of collecting these outcomes in a larger, powered study and conduct some exploratory analyses.

#### Group-level outcomes: RC

The RC survey is a seven-item instrument based on the RC framework [10, 11]. RC is measured between professional roles rather than between unique individuals [10]. Each RC dimension is summarized using a mean score, whereas the total RC index is a newly generated variable of a mean score for all seven dimensions [10, 11]. We will calculate RC scores for all participants (e.g., LPNs, RNs, physician residents, and physicians). A sample question is: How frequently do healthcare professionals in each of these groups communicate with you about patient care? See Additional file 1 for items and scoring. See Table 5 for the psychometric properties of study variables.

**Table 5:** Psychometric properties of study variables

Instrument	Number of items	Response format	α	Validity and factor analysis
Relational coordination	7	Varying response scale (Scores range from 7 to 35 and higher scores indicate greater RC)	0.8	Convergent exploratory factor analysis
Quality of care	3	Varying response scale (lower scores indicate higher quality of care)	–	–
Job satisfaction	3	1 single-item measure 2 open-ended questions	–	–
Intent to stay	4	1 = “strongly disagree,” 2 = “disagree,” 3 = “neither agree nor disagree,” 4 = “agree,” 5 = “strongly agree.”	0.85 (civilian participants) 0.91 (military participants)	Convergent and discriminant Exploratory factor analysis

#### Individual-level outcome: quality of care

Quality of care, defined as the extent to which healthcare services provided to individuals and patient populations improve desired health outcomes [30], will be explored from the healthcare professionals’ perception. Healthcare professionals view quality of care in terms of attributes of care, results of care, and the characteristics of patient and provider interactions [31]. We will explore the quality of care using three questions. These three questions demonstrated validity and reliability for use in a previous federally funded research study [32]. See Additional file 1.

#### Individual-level outcome: job satisfaction

Job satisfaction, defined as the extent to which people like their job [33], will be explored using three questions (one single-item measure and two open-ended questions). Single-item measures of job satisfaction show a strong correlation with multiple-item scales [34]. Thus, we selected a single-item measure to reduce participant response burden. We asked participants, “On the whole, how satisfied are you with your present job?” Responses range from “1 = very dissatisfied” to “5 = very satisfied,” with higher scores indicating better job satisfaction. See Additional file 1.

#### Individual-level outcome: intent to stay

Intent to stay, defined as the extent to which employees plan to continue working with their employer, will be measured using a 4-item intent to stay scale [33]. Responses range from “1 = strongly agree” to “5 = strongly disagree.” We will use reverse scoring where appropriate so that higher scale scores will indicate higher intent to stay. See Additional file 1 for items and scoring.

### Demographic variables

We will also collect data regarding participants’ demographic characteristics (race, sex, age, experience, education level, length of time worked on the unit, and certifications).

### Evaluations

We will use an RC knowledge assessment to explore participants’ understanding of RC at baseline and 2 weeks after each RC training intervention session. See Additional file 2 for items and scoring. Additionally, we will administer an RC training evaluation to explore participants’ experiences with the face-to-face RC training intervention. See Additional file 3.

### Data analysis

#### Quantitative analysis

Data spreadsheets will be imported into Stata (StataCorp, College Station, TX) [35]. Standard descriptive statistics (e.g., means and standard deviations for continuous variables such as age and percentage for categorical variables such as sex) will be used to summarize demographic variables. Acceptability will be assessed qualitatively (see the “ Qualitative analysis” section below).

Aim 1: Determine the feasibility of conducting an RC training intervention.

All feasibility outcomes will be summarized with descriptive statistics overall and by group. As this is a pilot study, the level of missing data will be documented, but imputation will not be performed.

Aim 2: To explore the acceptability of the RC training intervention.

### Qualitative analysis

*Focus group interviews*. Data analysis will be concurrent with data collection to monitor for data saturation. Participants’ responses will be transcribed verbatim, and two researchers will independently verify the accuracy of the Excel spreadsheet compared to the original Word file notes. Thematic analysis will be used to analyze qualitative interview data since it offers an organized method that allows the researcher to identify, analyze, and report themes [36]. Additionally, thematic analysis is a flexible method that offers the researcher the ability to focus on the meaning expressed by study participants and the identification of themes across interviews to produce an in-depth and rich account of the data [37]. The six steps of thematic analysis as described by Braun and Clark [36] are (1) familiarization of the data, (2) generation of codes, (3) combining codes into themes, (4) reviewing of themes, (5) determining the significance of themes, and (6) reporting of findings. The research team will initially read the transcripts to become familiar with the data. Transcripts will be re-read with the initial generation of codes. The research team will meet on a regular basis to discuss the codes being generated and to reach a consensus on any discrepancies. As the analysis progresses, the team will determine themes. Our approach to rigor will be guided by Morse et al. [38] who recommend verifying the data during the process of data analysis by “checking, confirming, making sure, and being certain” (p. 12). Reflexivity will be maintained through discourse regarding personal beliefs and judgments brought up by research team members in the process of analysis, with assumptions and interpretations verified by returning to the data [39]. See Additional file 4 for focus group interview questions.

Group-level and individual-level changes in RC.

We will also explore group-level changes in RC and the individual-level changes in quality of care, job satisfaction, and intent to stay among nurses and physicians following an RC training intervention). We will plot the individual changes over time using line graphs and estimate changes over time with point estimates and 95% confidence intervals. For RC, we will also create RC matrices and maps at each time point to further describe RC by professional role.

### Data monitoring and management

The research team will be responsible for data monitoring and ensuring that participants meet the inclusion criteria to participate in this study. Data from the online surveys will be stored on RedCAP, an encrypted and secure platform. Participants will scan a QR code to complete the surveys, and all surveys, comments, and annotations will be stored on RedCAP to ensure the data is properly protected from unauthorized users. We will name all files and folders by professional role and unit type. Files will be backed up twice during data collection from RedCAP to Stata to prevent accidental changes or partial/complete deletion of data or damage caused by computer viruses. A password and username will be required to access the data. The research team will be the only people who have access to the data.

### Reporting of adverse events

The research team does not expect that participants will experience adverse events given there is minimal risk related to this training intervention. However, the research team will continuously monitor participants for adverse events and unanticipated problems by physically observing the participants during the study (RC training intervention and focus group interviews). The principal investigator will report adverse events and unanticipated problems to the institutional review board. The research team will review the risk of adverse events and unanticipated problems weekly during data collection and we will adjust the protocol as needed. We will use the Common Terminology Criteria for Adverse Events v3.0 (CTCAE) to grade the severity of an adverse event.

### Confidentiality

A unique participant identification number will be assigned to participants who complete the RC surveys. The participant identification number will not include any personally identifiable information such as the participant’s name, employee identification number, or date of birth. Participant responses will remain confidential. Individual participant responses from the RC training intervention and focus group interviews will remain confidential and responses will only be shared with members of the research team.

## Discussion

To our knowledge, researchers have not explored an RC training intervention among healthcare professionals in a military hospital. In this pilot study, we will explore the feasibility and acceptability of an RC training intervention among healthcare professionals in an Army medical center. In previous studies, RC was associated with better patient outcomes (e.g., quality of care) [16] and better staff outcomes (e.g., job satisfaction and intent to stay) [17–21]. Enhancing the quality of communication and relationships among physicians and nurses through an RC training intervention may be a cost-effective way to improve patient and staff outcomes [17, 18]. We will refine the RC training intervention study based on the results and feedback we receive from participants. Additionally, we will disseminate the results of this study in peer-reviewed journals and at professional conferences. The results from this feasibility study are an important first step in testing the efficacy of an RC training intervention to improve patient and staff outcomes in military medical centers in a larger powered study.

In order to best deliver quality patient care, both military and non-military hospitals must better develop interprofessional teams through interprofessional education. A Cochrane systematic review of fifteen interprofessional education (IPE) [40] intervention studies indicate a range of effective healthcare outcomes, with an update in 2017 [41] of nine practice-based IPE interventions with similar findings. Both reviews concluded that the heterogeneity of interventions and outcomes as well as the quality of the evidence left many gaps in the effectiveness of IPE interventions [40, 41]. Exploring the interprofessional team’s communication and function through RC interventions is key to developing, testing, and offering effective clinical IPE educational interventions.

Testing the efficacy of an RC training intervention is important because the delivery of quality patient care is a core expectation for military and non-military hospitals. The vital role of interprofessional teams is conceptualized to be directly related to the quality of patient care. Developing, testing, and offering educational interventions to enhance interprofessional communication and function through RC is key. Additionally, researchers have found positive associations between favorable work environments, staff outcomes, and patient outcomes [42]. Developing a reproducible RC training intervention has the potential to improve healthcare environments. The expected outcomes of this feasibility pilot study include better communication between interprofessional teams, better work environments, and improved staff and patient outcomes alike.

There are some limitations to consider in conducting this study. The proposed study is a pilot to provide feasibility and acceptability data that will inform the intervention, recruitment, methods, and outcomes in the development of a subsequent study that will be powered to test the effectiveness of the intervention. The subject sample is not intended to be adequate to test statistical significance. Our pilot study is a complex intervention, requiring the assessment of multiple components to understand which components are related to the study outcomes. Although health systems have lower COVID surges and admissions, the clinical practice arena has not yet recovered which could lead to barriers in recruiting participants, access to hospital units, or delays in conducting the study.

## Conclusion

In conclusion, upon IRB approval we will conduct a single-group feasibility pilot on three units in an Army medical center in the southeast USA. A convenience sample of LPNs, RNs, resident physicians, and physicians from three units (intensive care unit, medical-surgical, and labor and delivery unit) will be invited to complete a 1-h RC training intervention once a month over 3 months. The findings from this study will establish the feasibility and acceptability of conducting an RC intervention in a military hospital and inform refinement of the intervention and study procedures prior to conducting a larger randomized controlled trial to establish efficacy.

## Supplementary Information

Additional file 1. Participant survey.

Additional file 2. RC knowledge assessment.

Additional file 3. Relational Coordination Training Evaluation: Improving Quality of Care, Job Satisfaction, and Intent to Stay.

Additional file 4. Focus group interview questions

## Data Availability

The datasets generated and/or analyzed will be available from the corresponding author upon request.

## Abbreviations

RC: Relational coordination
IPE: Interprofessional education
