# Supplementary material for: A pilot study protocol of a relational coordination training intervention among healthcare professionals in an Army medical center
Source: Pilot Feasibility Stud. 2025 Mar 4;11:25. doi: 10.1186/s40814-025-01596-7 (PMC11877811; doi:10.1186/s40814-025-01596-7)
Supplement: Supplementary file 1 — Additional file 1. Participant survey. [file 40814_2025_1596_MOESM1_ESM.docx]

**Additional file 1.** Participant survey

*When answering the relational coordination questions, be sure to consider all forms of communication, including in-person meetings, phone calls, e-mails, etc. Select the Not Applicable (N/A) answer choice if interaction with the workgroup/individual listed is not needed with your role or if you do not wish to answer this question.*

1. **Frequent Communication**

*How* ***frequently*** *do people in each of these groups communicate with you about the* ***patient care****?*

| **Licensed Practical Nurses (LPNs)** | Not Nearly Enough   | Not  Enough   | Just the Right Amount   | Too Often   | Much Too Often   | Not  Applicable   |
| --- | --- | --- | --- | --- | --- | --- |
| **Registered Nurses (RNs)** | Not Nearly Enough   | Not  Enough   | Just the Right Amount   | Too Often   | Much Too Often   | Not  Applicable   |
| **Resident Physicians** | Not Nearly Enough   | Not  Enough   | Just the Right Amount   | Too Often   | Much Too Often   | Not  Applicable   |
| **Physicians** | Not Nearly Enough   | Not  Enough   | Just the Right Amount   | Too Often   | Much Too Often   | Not  Applicable   |

1. **Timely Communication**

*Do they communicate with you in a* ***timely*** *way about* ***patient care?***

| **Licensed Practical Nurses (LPNs)** | Never   | Rarely   | Sometimes   | Often   | Always   | Not  Applicable   |
| --- | --- | --- | --- | --- | --- | --- |
| **Registered Nurses (RNs)** | Never   | Rarely   | Sometimes   | Often   | Always   | Not  Applicable   |
| **Resident Physicians** | Never   | Rarely   | Sometimes   | Often   | Always   | Not  Applicable   |
| **Physicians** | Never   | Rarely   | Sometimes   | Often   | Always   | Not  Applicable   |

1. **Accurate Communication**

*Do they* ***communicate*** *with you accurately about the* ***patient care?***

| **Licensed Practical Nurses (LPNs)** | Never   | Rarely   | Sometimes   | Often   | Always   | Not  Applicable   |
| --- | --- | --- | --- | --- | --- | --- |
| **Registered Nurses (RNs)** | Never   | Rarely   | Sometimes   | Often   | Always   | Not  Applicable   |
| **Resident Physicians** | Never   | Rarely   | Sometimes   | Often   | Always   | Not  Applicable   |
| **Physicians** | Never   | Rarely   | Sometimes   | Often   | Always   | Not  Applicable   |

1. **Problem-Solving Communication**

*When there is a problem with the* ***patient care,*** *do people in each of these groups blame others or work with you to* ***solve*** *the problem?*

| **Licensed Practical Nurses (LPNs)** | Always Blame   | Mostly Blame   | Neither Blame nor Solve   | Mostly  Solve   | Always Solve   | Not  Applicable   |
| --- | --- | --- | --- | --- | --- | --- |
| **Registered Nurses (RNs)** | Always Blame   | Mostly Blame   | Neither Blame nor Solve   | Mostly  Solve   | Always Solve   | Not  Applicable   |
| **Resident Physicians** | Always Blame   | Mostly Blame   | Neither Blame nor Solve   | Mostly  Solve   | Always Solve   | Not  Applicable   |
| **Physicians** | Always Blame   | Mostly Blame   | Neither Blame nor Solve   | Mostly  Solve   | Always Solve   | Not  Applicable   |

1. **Shared Goals**

*Do people in each of these groups* ***share your goals*** *for* ***patient care****?*

| **Licensed Practical Nurses (LPNs)** | Not At All   | A Little   | Somewhat   | A Lot   | Completely   | Not  Applicable   |
| --- | --- | --- | --- | --- | --- | --- |
| **Registered Nurses (RNs)** | Not At All   | A Little   | Somewhat   | A Lot   | Completely   | Not  Applicable   |
| **Resident Physicians** | Not At All   | A Little   | Somewhat   | A Lot   | Completely   | Not  Applicable   |
| **Physicians** | Not At All   | A Little   | Somewhat   | A Lot   | Completely   | Not  Applicable   |

1. **Shared Knowledge**

*Do people in each of these groups* ***know*** *about the work you do with* ***patient care****?*

| **Licensed Practical Nurses (LPNs)** | Nothing   | A Little   | Some   | A Lot   | Everything   | Not  Applicable   |
| --- | --- | --- | --- | --- | --- | --- |
| **Registered Nurses (RNs)** | Nothing   | A Little   | Some   | A Lot   | Everything   | Not  Applicable   |
| **Resident Physicians** | Nothing   | A Little   | Some   | A Lot   | Everything   | Not  Applicable   |
| **Physicians** | Nothing   | A Little   | Some   | A Lot   | Everything   | Not  Applicable   |

1. **Mutual Respect**

Do people in each of these groups ***respect*** the work you do with ***patient care****?*

| **Licensed Practical Nurses (LPNs)** | Not At All   | A Little   | Somewhat   | A Lot   | Completely   | Not  Applicable   |
| --- | --- | --- | --- | --- | --- | --- |
| **Registered Nurses (RNs)** | Not At All   | A Little   | Somewhat   | A Lot   | Completely   | Not  Applicable   |
| **Resident Physicians** | Not At All   | A Little   | Somewhat   | A Lot   | Completely   | Not  Applicable   |
| **Physicians** | Not At All   | A Little   | Somewhat   | A Lot   | Completely   | Not  Applicable   |

**Quality of Care**

Please answer the following questions.

1. In general, how would you describe the quality of care delivered to patients on your unit?

1. Excellent 2. Good 3. Fair 4. Poor

2. How would you describe the quality of care delivered during your last shift?

1. Excellent 2. Good 3. Fair 4. Poor

3. Overall, over the past year would you say the quality of patient care in your hospital has:

1. Improved 2. Remained the same 3. Deteriorated

**Job Satisfaction**

1. On the whole, how satisfied are you with your present job?

| Very Dissatisfied   | Dissatisfied   | Neutral   | Satisfied   | Very Satisfied   |
| --- | --- | --- | --- | --- |

1. In what areas do you find the most satisfaction with your work?
2. In what areas do you find the least satisfaction with your work?

**Intent to Stay (Civilian Participants)**

*Please answer the following questions.*

1. I plan to leave this hospital as soon possible.

| Strongly Agree   | Agree   | Neither Agree nor Disagree   | Disagree   | Strongly Disagree   |
| --- | --- | --- | --- | --- |

1. Under no circumstances will I voluntarily leave this hospital.

| Strongly Agree   | Agree   | Neither Agree nor Disagree   | Disagree   | Strongly Disagree   |
| --- | --- | --- | --- | --- |

1. I will be reluctant to leave this hospital.

| Strongly Agree   | Agree   | Neither Agree nor Disagree   | Disagree   | Strongly Disagree   |
| --- | --- | --- | --- | --- |

1. I plan to stay at this hospital as long as possible.

| Strongly Agree   | Agree   | Neither Agree nor Disagree   | Disagree   | Strongly Disagree   |
| --- | --- | --- | --- | --- |

**Intent to Stay (Military Participants)**

*Please answer the following questions:*

1. I plan to leave the Army as soon possible.

| Strongly Agree   | Agree   | Neither Agree nor Disagree   | Disagree   | Strongly Disagree   |
| --- | --- | --- | --- | --- |

1. Under no circumstances will I voluntarily leave the Army.

| Strongly Agree   | Agree   | Neither Agree nor Disagree   | Disagree   | Strongly Disagree   |
| --- | --- | --- | --- | --- |

1. I will be reluctant to leave the Army.

| Strongly Agree   | Agree   | Neither Agree nor Disagree   | Disagree   | Strongly Disagree   |
| --- | --- | --- | --- | --- |

1. I plan to stay in the Army as long as possible.

| Strongly Agree   | Agree   | Neither Agree nor Disagree   | Disagree   | Strongly Disagree   |
| --- | --- | --- | --- | --- |

**Demographic Questions**

*Please answer the following questions.*

1. What is your age in years?
2. What is your race?

| Asian   | Black/African American   | American Indian   | Caucasian   | Hispanic   | Other   |
| --- | --- | --- | --- | --- | --- |

1. What is your sex?

| Female   | Male   | Non-binary   |
| --- | --- | --- |

1. Which hospital unit do you work on?
2. What is your professional role?

| Licensed Practical Nurse (LPN) |  |
| --- | --- |
| Registered Nurse (RN) |  |
| Resident Physician |  |
| Physician |  |

1. What is your highest completed degree?

| Diploma   | Associate Degree   | Baccalaureate Degree   | Graduate Degree   | Doctoral Degree   |
| --- | --- | --- | --- | --- |

1. Please list any credentials you have related to your job role.
2. How long have you been in your career (e.g., How long have you been a nurse or physician)?
3. How long have you worked at this hospital?
4. How long have you worked on this unit?
