# Supplementary material for: A pilot study protocol of a relational coordination training intervention among healthcare professionals in an Army medical center
Source: Pilot Feasibility Stud. 2025 Mar 4;11:25. doi: 10.1186/s40814-025-01596-7 (PMC11877811; doi:10.1186/s40814-025-01596-7)
Supplement: Supplementary file 2 — Additional file 2. RC knowledge assessment. [file 40814_2025_1596_MOESM2_ESM.docx]

**Additional file 2**. RC knowledge assessment

**Instructions**: Please answer the following questions below.

1. On a scale of 1-10, please rate **your** **current level of knowledge** about relational coordination:

| 1 | 2 | 3 | 4 | 5 | 6 | 7 | 8 | 9 | 10 |
| --- | --- | --- | --- | --- | --- | --- | --- | --- | --- |
|  |  |  |  |  |  |  |  |  |  |

No knowledge at all (1) Some knowledge (5) Expert knowledge (10)

1. On a scale of 1-10, please rate how **comfortable** you would be using relational coordination to improve the patient care process:

| 1 | 2 | 3 | 4 | 5 | 6 | 7 | 8 | 9 | 10 |
| --- | --- | --- | --- | --- | --- | --- | --- | --- | --- |
|  |  |  |  |  |  |  |  |  |  |

Not comfortable at all (1) Somewhat comfortable (5) Completely comfortable (10)

*Note. Questions 1 (perceived knowledge) and 2 (comfort) will be analyzed separately as individual items.*

Please select the **BEST** answer.

1. What is relational coordination?
2. Relational coordination is a validated measure of teamwork.
3. “A mutually reinforcing process of communicating and relating for the purpose of task integration within and between roles.”
4. “Organizing patient care activities and sharing information among all healthcare professionals involved with patient care.”
5. Both A & B.
6. Both A & C.
7. **Select all that apply**: Relational coordination encompasses the following communication dimensions?
8. Frequent communication
9. Timely communication
10. Problem-solving communication
11. Accurate communication
12. Relational communication
13. **Select the best answer:** Relational coordination encompasses the following relationship dimensions:
14. Shared goals
15. Shared knowledge
16. Relational care coordination
17. Mutual respect
18. A, B, & D
19. All the above

Questions #6-7 pertain to the following case. Please respond by selecting the BEST answer.

RN Rita is eating lunch in the breakroom with RN Jose. RN Rita states, “I feel so overwhelmed today and my patients are very sick. Staffing has been horrible on our unit, and I feel like we do not have enough support. I sent our nurse manager an email last week expressing my concerns, but I have not received a response. I feel so devalued working here.”

1. **Select all that apply**. What dimensions of relational coordination can RN Jose use to address RN Rita’s concerns about her experience with the nurse manager?
2. Problem-solving communication
3. Shared goals
4. Relational communication
5. Shared knowledge
6. Frequent communication
7. **(Open ended question: Please write your response)** How should RN Jose respond to RN Rita’s concerns?
