# Supplementary material for: A pilot study protocol of a relational coordination training intervention among healthcare professionals in an Army medical center
Source: Pilot Feasibility Stud. 2025 Mar 4;11:25. doi: 10.1186/s40814-025-01596-7 (PMC11877811; doi:10.1186/s40814-025-01596-7)
Supplement: Supplementary file 3 — Additional file 3. Relational Coordination Training Evaluation: Improving Quality of Care, Job Satisfaction, and Intent to Stay. [file 40814_2025_1596_MOESM3_ESM.docx]

**Additional file 3.**

Relational Coordination Training Evaluation: Improving Quality of Care, Job Satisfaction, and Intent to Stay

**Facilitator** Sherita House

**Date**

*Please rate your level agreement with each of the following statements about outcomes of this intervention workshop.*

|  | **Not at all** | **A little** | **Some** | **A lot** | **Totally** |
| --- | --- | --- | --- | --- | --- |
| I have a deeper understanding of relational coordination and its importance for performance outcomes. | 1 | 2 | 3 | 4 | 5 |
| I am able to use relational mapping to generate new ideas for how I communicate and relate to other healthcare professionals regarding patient care. | 1 | 2 | 3 | 4 | 5 |
| This intervention training was a worthwhile experience. | 1 | 2 | 3 | 4 | 5 |
| As a result of this training, I have seen improvements in quality of care on this unit. | 1 | 2 | 3 | 4 | 5 |
| As a result of this training, I have seen improvements in my job satisfaction. | 1 | 2 | 3 | 4 | 5 |
| As a result of this training, I have higher intent to stay at this hospital. | 1 | 2 | 3 | 4 | 5 |

What did you appreciate most about the relational coordination training intervention?

Please describe a moment in this training that stands out for you as meaningful or useful.

How can this intervention training be improved?

Please feel free to offer any feedback you have for the facilitator.

Thank you!
