# Supplementary material for: A pilot study protocol of a relational coordination training intervention among healthcare professionals in an Army medical center
Source: Pilot Feasibility Stud. 2025 Mar 4;11:25. doi: 10.1186/s40814-025-01596-7 (PMC11877811; doi:10.1186/s40814-025-01596-7)
Supplement: Supplementary file 4 — Additional file 4. Focus group interview questions [file 40814_2025_1596_MOESM4_ESM.docx]

**Additional file 4.** Focus group interview questions

1. How has the relational coordination training intervention impacted the quality of care on your unit?
2. How has the relational coordination training intervention impacted job satisfaction on your unit?
3. How has the relational coordination training intervention impacted intent to stay on your unit?
4. Which relational coordination dimensions have been the most effective in improving quality of care? What made these dimensions the most effective?
5. Which relational coordination dimensions have been the most effective in improving job satisfaction? What made these dimensions the most effective?
6. Which relational coordination dimensions have been the most effective in improving intent to stay? What made these dimensions the most effective?
7. Which relational coordination dimensions have been the least effective (e.g., problematic) in improving quality of care? What made these dimensions the least effective?
8. Which relational coordination dimensions have been the least effective (e.g., problematic) in improving job satisfaction? What made these dimensions the least effective?
9. Which relational coordination dimensions have been the least effective (e.g., problematic) in improving intent to stay? What made these dimensions the least effective?
10. How did you experience relational coordination with other healthcare professionals (e.g., nurses, physician residents, and physicians) involved in the patient care process?
11. Did you experience any barriers in completing the relational coordination training intervention? If so, what barriers did you experience?
12. What improvements can be made to enhance this relational coordination training intervention?
